# Barriers and facilitators to adoption of soft-copy interpretation from the user perspective: A comment

**DOI:** 10.4103/2153-3539.77171

**Published:** 2011-02-26

**Authors:** Viroj Wiwanitkit

**Affiliations:** Wiwanitkit House, Bangkhae, Bangkok 10160, Thailand

Sir,

I read the recent publication on ‘Barriers and facilitators to adoption of soft-copy interpretation from the user perspective’ with a great interest.[1] Patterson *et al*. concluded that ‘improving performance using digital images in pathology would likely accelerate adoption of innovative technologies that are facilitated by the use of digital images, such as electronic imaging databases, electronic health records, double reading for challenging cases, and computer-aided diagnostic systems.[1] I would like to make a few observations with regard to this article. First, this work used a non-standard interview format, with a very long questionnaire (requiring a 1-hour period) covering very few subjects. The outcomes may not be generalizable. Second, if we wish to trace the barriers for assessment, it might be better to compare groups that have no accessibility to the ones that have. Indeed, the group selection might have an impact on the assessment. If one wishes to know what the barriers are, one should select the exact negative group (with the exact opposite characterisitics) to be the control group for the study.
